# A Practical Treatise on Phthisis Pulmonalis

**Published:** 1861-03

**Authors:** 


A Practical Treatise on Phthisis Pulmonalis; containing its Pathology, Causes, Symptoms and Treatment. By L. M. Lawson, M. D., Professor of Clinical Medicine in the University of Louisiana. Rickey, Mallory & Co. Cincinnati: S. S. & W. Wood, New York. From the Cincinnati Publishers.

This is a well bouud octavo volume of 557 pages. From the well known, and deserved reputation of the author, together with a hasty glance at the contents of the work, we do not hesitate to recommend it as worthy of a place in every physician's library. We shall give it a more extended notice when we have had time to examine it with the necessary care.

